# Inhibitory Effect of *β*-Sitosterol on the Ang II-Induced Proliferation of A7r5 Aortic Smooth Muscle Cells

**DOI:** 10.1155/2023/2677020

**Published:** 2023-11-07

**Authors:** Yuankun Chen, Shumiao He, Ao Zeng, Siqing He, Xiaobao Jin, Chunmei Li, Wenjie Mei, Qun Lu

**Affiliations:** ^1^School of Basic Medical Sciences, Guangdong Pharmaceutical University, No. 280 East Outer Ring Road, Panyu District, Guangzhou, China; ^2^Department of Infectious and Tropical Diseases, The Second Affiliated Hospital of Hainan Medical University, Haikou, China; ^3^Guangdong Province Key Laboratory of Pharmaceutical Bioactive Substances, Guangdong Pharmaceutical University, Guangzhou, China; ^4^Guangdong Province Engineering and Technology Center for Molecular Probe and Bio-medicine Imaging, Guangzhou, China

## Abstract

**Objective:**

To explore the effects of *β*-sitosterol on VSMC proliferation.

**Materials and Methods:**

A7r5 cells were pretreated with 2 *µ*M angiotensin II (Ang II) for 24 hr to establish an excessive VSMC proliferation model, followed by treatment with *β*-sitosterol for 24 hr. Cells were divided into five groups: control, Ang II, and Ang II + *β*-sitosterol (2, 4, 8 *µ*M). CCK-8 assay, flow cytometry, and Ad-mCherry-GFP-LC3B assay analyzed cell proliferation, cell cycle, cell apoptosis, and autophagic flux. Additionally, the expression of proteins was detected by the western blotting.

**Results:**

*β*-Sitosterol effectively inhibited Ang II-induced A7r5 cell proliferation (IC50 : 6.841 *µ*M at 24 hr). It achieved this by arresting cell cycle progression, promoting apoptosis, inhibiting autophagy, and suppressing the contractile–synthetic phenotypic switch. Mechanistically, *β*-sitosterol downregulated PCNA, Cyclin D1, and Bcl-2, while upregulating pro-caspase 3, cleaved-caspase 3, and Bax to induce cell cycle arrest and apoptosis. Additionally, it suppressed the contractile–synthetic phenotypic transformation by downregulating OPN and upregulating *α*-SMA. The Ad-mCherry-GFP-LC3B Assay and western blotting revealed *β*-sitosterol's autophagy inhibitory effects by downregulating LC3, ULK1, and Beclin-1 while upregulating P62 expression. *Discussion and Conclusion*. This study found for the first time that *β*-sitosterol could inhibit the proliferation of A7r5 cells induced by Ang II. *β*-Sitosterol treatment may be recommended as a therapeutic strategy to prevent the cardiovascular diseases.

## 1. Introduction

Vascular smooth muscle cells (VSMCs), a critical component of tunica media, play an irreplaceable role in regulating vascular tone, allowing vascular remodeling, and providing structural support [1–3]. Excessive proliferation of VSMCs contributes to cardiovascular diseases, including atherosclerosis, aneurysms, and intimal restenosis [4, 5]. Thus, the development of novel agents to inhibit the excessive proliferation of VSMCs is urgently required to prevent cardiovascular diseases.

Various cytokines and growth factors, including insulin, angiotensin II (Ang II), and platelet-derived growth factors, stimulate excessive proliferation of VSMCs [6–8]. Ang II, an important peptide in the renin–angiotensin system, stimulates the proliferation of VSMCs by regulating cell cycle progression and apoptosis [9]. A recent study found that Ang II-induced proliferation of VSMCs was related to the regulation of autophagy [10].

Phytosterols are natural plant components structurally similar to cholesterol and are enriched in traditional Chinese herbal medicines, vegetable oils, and plant seeds [11, 12]. The previous studies have shown that phytosterols and their derivatives protect the cardiovascular system by reducing the absorption of dietary cholesterol [13–15]. Animal studies have shown that feeding mice with 2% mixed phytosterols can reduce tumor size, inhibit cancer cell growth, and significantly reduce the incidence of prostate, breast, and colon cancer [16, 17]. In vitro studies have also demonstrated that phytosterols, particularly *β*-sitosterol, could inhibit the proliferation and induce apoptosis in osteoblasts and various cancer cells [18–20]. Furthermore, stigmasterol can interrupt the progression of the cell cycle in rat aortic smooth muscle cells (RASMCs) [9]. However, the effect of *β*-sitosterol on VSMC proliferation remains unknown. Thus, we studied the effect of *β*-sitosterol on the proliferation of A7r5 model cell lines induced by Ang II and explored the related mechanisms.

## 2. Materials and Methods

### 2.1. Reagents

*β*-Sitosterol (98%, MB6654) was purchased from Dalian Meilune Biotechnology Co., Ltd. (Dalian, China). Ang II (05-23-0101-1MGCN) was obtained from Merck Millipore (Darmstadt, Germany). Cell Counting Kit-8 (CCK-8; C0037), Ad-mCherry-GFP-LC3B (C3011), Z-VAD-FMK (C1202), and Annexin V-FITC Kit (C1062S) were procured from Beyotime Biotechnology Co., Ltd. (Nantong, China). Fetal bovine serum (FBS; 16000044), phosphate-buffered saline (PBS; 10010031), and Dulbecco's Modified Eagle Medium (DMEM; 11965084) were obtained from Gibco (Gaithersburg, MD, USA). A Chemiluminescence Detection Kit (RPN2106) was purchased from Amersham Biosciences (Buckinghamshire, UK). Anti-caspase 3 antibody (ab49822), anti-Bax antibody (ab32503), anti-Bcl-2 antibody (ab194583), anti-P62 antibody (ab109012), anti-OPN antibody (ab214050), anti-*α*-SMA antibody (ab5694), anti-LC3B antibody (ab192890), anti-PCNA antibody (ab92552), anti-Cyclin D1 antibody (ab40754), anti-Beclin 1 antibody (ab210498), and the secondary antibody (ab6721) were obtained from Abcam (Burlingame, CA, USA). Anti-ULK1 antibody (8054) was purchased from Cell Signal Technology (Boston, MA, USA).

### 2.2. Cell Culture and Treatment

The A7r5 cells were procured from the Cell Bank of the Chinese Academy of Sciences (Shanghai, China). A7r5 cells were seeded in DMEM containing 1% penicillin/streptomycin and 10% FBS and cultured at 37°C in an incubator with 5% CO_2_. Third-to-sixth generation A7r5 cells were used in this experiment. After reaching 70%–80% confluence, A7r5 cells were washed with PBS and cultured in a medium with and without Ang II and *β*-sitosterol at different concentrations for 6–24 hr. Concentrations of *β*-sitosterol and Ang II were determined as described previously [20, 21].

### 2.3. Assessment of Cytotoxicity and Cell Proliferation via CCK-8 Assay

The CCK-8 was used to evaluate the cytotoxicity of *β*-sitosterol in A7r5 cells. The assay was repeated five times. A7r5 cells were seeded in 96-well plates at a density of 5 × 10^3^ cells/well at room temperature. After cell culture overnight, each well of A7r5 cells was cultured in a medium with and without Ang II (2 *µ*M) and *β*-sitosterol (0.5, 1, 2, 4, 8 *µ*M) for 24 hr. Subsequently, 10 *µ*l WST-8 solution was added and cultured in an incubator for 1–2 hr, and A7r5 cells in 96-well plates were detected using a microplate absorbance reader at 450 nm.

### 2.4. Assessment of Cell Cycle and Cell Apoptosis via Flow Cytometry

The cell cycle of A7r5 cells was determined using flow cytometry. In brief, cells were seeded into 6-well plates and then fixed in 70% cold ethanol following treatment with Ang II or *β*-sitosterol. Cells were then washed with PBS, resuspended in PBS containing ribonuclease, and then stained with propidium iodide in the dark. The fluorescence intensity of the cells (1 × 10^4^ cells in each sample) was quantified by the flow cytometer (FACSCalibur™ cytometer, BD Biosciences, USA).

A7r5 cells were assessed for apoptosis using the Annexin V-FITC Kit in accordance with the manufacturer's instructions. A7r5 cells were trypsinized, washed twice with cold PBS, resuspended in 500 *μ*l binding buffer containing 5 *μ*l annexin V-FITC and 5 *μ*l PI, and cultured at room temperature in the dark for 15 min. Finally, cell apoptosis was detected using flow cytometer (FACSCalibur™ cytometer, BD Biosciences, USA).

### 2.5. Assessment of Autophagic Flux via Ad-mCherry-GFP-LC3B Assay

Induction of autophagic flux was assessed using the Ad-mCherry-GFP-LC3B assay. A7r5 cells were seeded in 6-well culture plates at a density of 2 × 10^4^ cells per well until the cells reached 40–50% confluency. Cells were transfected with Ad-mCherry-GFP-LC3B at an MOI of 20 for 24 hr according to the protocol. After that, the cells were treated with *β*-sitosterol, Ang II or 3-MA for another 24 hr and their morphology was observed under a fluorescence microscope (Leica, Germany).

### 2.6. Western Blot

Treated A7r5 cells were lysed in lysis buffer (Tris-HCl, NaCl, EDTA, 10% glycerol, 0.1% NP-40, PMSF, aprotinin, leupeptin, NaF, Na3VO4, and DTT) and incubated for 30 min on ice. The supernatant was collected by centrifugation at 12,000 rpm for 10 min at 4°C. A BCA Protein Assay Kit was used to determine the total protein concentration in the supernatant. Proteins from A7r5 cells were separated by 15% sodium dodecyl sulfate-polyacrylamide gel electrophoresis and were transferred onto PVDF membranes. The PVDF membranes were blocked with 5% nonfat milk for 2 hr and incubated with primary antibodies overnight at 4°C, followed by goat anti-rabbit secondary antibodies for 2 hr at room temperature. The PVDF membranes were visualized using a chemiluminescence detection kit, and quantitative analyzes were performed by densitometry using ImageJ 6.0 software (National Institute of Health, Bethesda, Maryland, USA).

### 2.7. Statistical Analysis

Data are shown as mean ± SD. Significant differences among multiple groups were determined using one-way analysis of variance (ANOVA) and Dunnett's test with GraphPad Prism 7.1 software (GraphPad Software, San Diego, CA, USA). Statistical significance was set at *P* < 0.05.

## 3. Result

### 3.1. Effects of Ang II and *β*-Sitosterol on the Viability of A7r5 Cells

**Figure 1:**
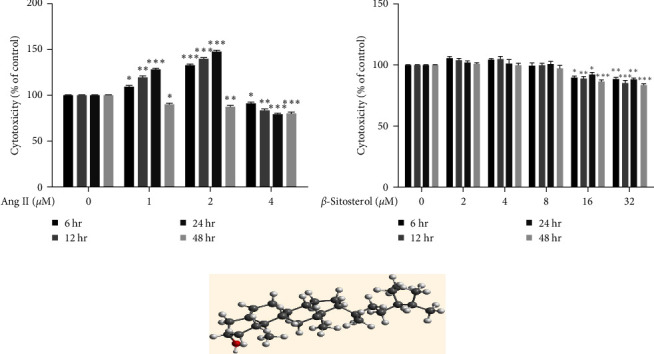
Effect of Ang II and *β*-sitosterol on A7r5 cell viability. (a) The cytotoxic effect of Ang II on A7r5 cells was detected using the CCK-8 assay. (b) The cytotoxic effect of *β*-sitosterol on A7r5 cells was detected using the CCK-8 assay. (c) 3D chemical structure of *β*-sitosterol. Data indicate the mean ± SD (*n* = 5, *n*: number of the sample). Significant differences in different groups compared with the control group (without treatment with Ang II or *β*-sitosterol) are expressed as  ^*∗*^*P* < 0.05,  ^*∗*^ ^*∗*^*P* < 0.01, and  ^*∗*^ ^*∗*^ ^*∗*^*P* < 0.001.

To study the effect of *β*-sitosterol on Ang II-induced A7r5 cells, we treated A7r5 cells with Ang II (0, 1, 2, and 4 *µ*M) and *β*-sitosterol (0, 2, 4, 8, 16, and 32 *µ*M) for 6, 12, 24, or 48 hr to determine cell viability and select the appropriate doses of Ang II and *β*-sitosterol. Cell viability was determined using a CCK-8 assay. As shown in Figure 1(a), Ang II (1 and 2 *µ*M) significantly increased the proliferation of A7r5 cells at 6, 12, and 24 hr, but not at 48 hr. Compared with the control group, 1 *µ*M Ang II significantly increased A7r5 cell growth by 9% (*P* < 0.05), 19% (*P* < 0.01), and 28% (*P* < 0.001) at 6, 12, and 24 hr, respectively. However, it decreased cell proliferation by 10% (*P* < 0.05) and showed obvious cytotoxicity at 48 hr. Therefore, 48 hr of Ang II treatment was not used for further experiments. Moreover, A7r5 cells treated with 2 *µ*M Ang II for 24 hr showed the highest proliferation rate. Therefore, 2 *µ*M and 24 hr were chosen as the treatment dose and time, respectively, for Ang II in A7r5 cells for further study. As shown in Figure 1(b), *β*-sitosterol at concentrations of 2, 4, and 8 *µ*M exerted no cytotoxicity on A7r5 cells. The 3D chemical structure of *β*-sitosterol was shown in Figure 1(c).

### 3.2. Inhibitory Effect of *β*-Sitosterol on the Ang II-Induced Proliferation of A7r5 Cells

**Figure 2:**
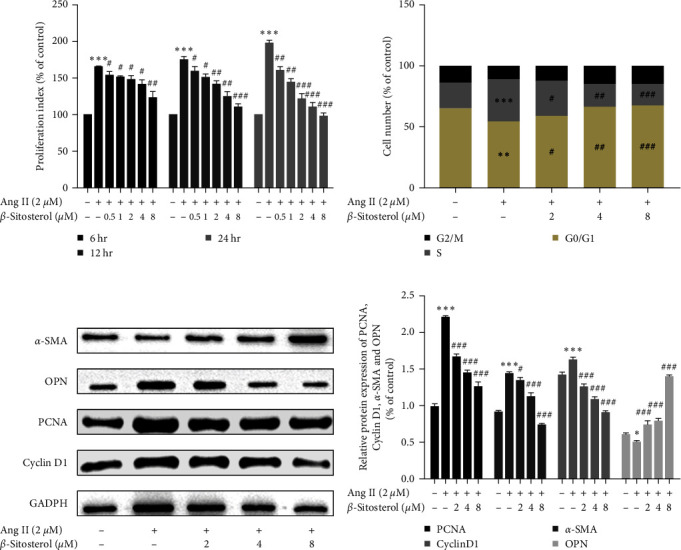
Effects of *β*-sitosterol on the proliferation, cell cycle progression, and phenotypic switch of Ang II-induced A7r5 cells. (a) Effects of *β*-sitosterol on the proliferation of Ang II-induced A7r5 cells were detected using the CCK-8 assay. (b) The cell cycle progress of A7r5 cells was tested by flow cytometry. (c) Protein expression of *α*-SMA, OPN, PCNA, and Cyclin D1 was examined by western blotting. (d) *α*-SMA, OPN, PCNA and Cyclin D1 protein development gray value analysis histogram. Data indicate the mean ± SD (*n* = 5, *n*: number of the sample). Significant differences in different groups compared with the control group (without treatment with Ang II or *β*-sitosterol) are expressed as  ^*∗*^*P* < 0.05,  ^*∗*^ ^*∗*^*P* < 0.01,  ^*∗*^ ^*∗*^ ^*∗*^*P* < 0.001. Significant differences in different groups compared with the Ang II-alone treatment group are expressed as ^#^*P* < 0.05, ^###^*P* < 0.01, ^###^*P* < 0.001.

To determine whether *β*-sitosterol could inhibit the Ang II-induced excessive proliferation of A7r5 cells, we pretreated A7r5 cells with 2 *µ*M Ang II before treatment with *β*-sitosterol (0.5, 1, 2, 4, and 8 *µ*M). The proliferation index of A7r5 cells was examined using the CCK-8 assay. The results showed that the proliferation index of the Ang II-alone treatment group was greater than that of the control group (*P* < 0.001) (Figure 2(a)). The proliferation index of Ang II-induced A7r5 cells decreased over time (6, 12, and 24 hr) and in a concentration-dependent manner (0.5, 1, 2, 4, and 8 *µ*M) after *β*-sitosterol treatment (Figure 2(a)). The half-maximal inhibition concentration (IC_50_) of *β*-sitosterol-treated Ang II-induced A7r5 cells at 6, 12, or 24 hr was 48.1, 17.76, and 6.841 *µ*M, respectively. Therefore, 2, 4, and 8 *µ*M were used as the concentrations of *β*-sitosterol for subsequent experiments. The above results indicate that *β*-sitosterol could inhibit the excessive proliferation of Ang II-induced A7r5 cells.

### 3.3. Inhibitory Effect of *β*-Sitosterol on Ang II-Induced Contractile–Synthetic Phenotypic Switch in A7r5 Cells

The contractile and synthetic phenotypes are two types of VSMCs. The transformation from the contractile phenotype to the synthetic phenotype results in VSMC proliferation, which leads to hypertension. We first examined changes in the expression of cell contractile phenotypic marker *α*-actin (*α*-SMA) and synthetic phenotypic marker osteopontin (OPN) in A7r5 cells by western blotting. Compared with the control group, we observed a downregulation in the expression level of *α*-SMA and an upregulation in that of OPN in the Ang II-alone treatment group. However, treatment with *β*-sitosterol increased *α*-SMA expression and decreased OPN expression compared to the Ang II-alone treatment group (Figures 2(c) and 2(d)). These results suggest that *β*-sitosterol inhibits the Ang II-induced phenotype switching from a contractile to synthetic state in A7r5 cells.

### 3.4. Cell Cycle Arrest in Ang II-Induced A7r5 Cells by *β*-Sitosterol

Cell cycle progression was examined by flow cytometry to confirm whether *β*-sitosterol could inhibit the proliferation of Ang II-induced A7r5 cells. As shown in Figure 2(b), treatment of A7r5 cells with Ang II decreased the proportion of cells in the G0/G1 phase from 65.74% ± 1.02% to 54.89% ± 1.43% (*P* < 0.01) and increased the proportion of cells in the S phase from 19.93% ± 1.46% to 33.49% ± 1.34% (*P* < 0.001) compared to the control group. The expression levels of PCNA and Cyclin D1 were also examined. PCNA and Cyclin D1 expression levels were found significantly upregulated in Ang II-alone treatment groups (Figures 2(c) and 2(d)). However, treatment with 2, 4, and 8 *µ*M of *β*-sitosterol increased the proportion of A7r5 cells in the G0/G1 phase to 58.70% ± 1.88% (*P* < 0.05), 67.20% ± 2.39% (*P* < 0.01), and 67.74% ± 1.97% (*P* < 0.001), and decreased the proportion of A7r5 cells in the S phase to 28.85% ± 2.25% (*P* < 0.05), 17.70% ± 1.84% (*P* < 0.01), and 17.13% ± 3.11% (*P* < 0.001) compared with the Ang II-alone treatment group. Additionally, the expression levels of PCNA and Cyclin D1 were significantly downregulated in the *β*-sitosterol treatment groups compared with those in the Ang II-alone treatment group. The proportion of A7r5 cells in the G2/M phase in the Ang II alone treatment group and the *β*-sitosterol treatment group did not differ from that in the control group. These results indicated that *β*-sitosterol increased the proportion of Ang II-induced A7r5 cells in the G0/G1 phase and decreased the proportion of these cells in the S phase.

### 3.5. Enhancing Effect of *β*-Sitosterol on the Apoptosis of Ang II-Induced A7r5 Cells

**Figure 3:**
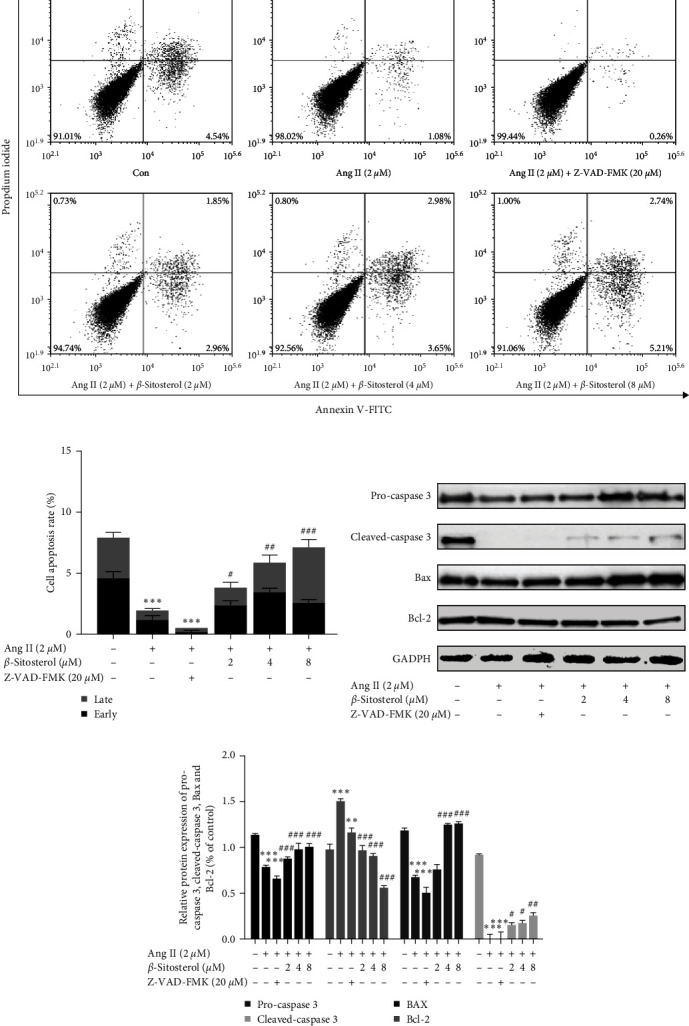
Effect of *β*-sitosterol on the apoptosis of Ang II-induced A7r5 cells. (a) A7r5 cells were treated, and apoptosis of A7r5 cells was examined by flow cytometry. (b) Statistical histogram of early and late cell apoptosis rate. (c) Protein expression of pro-caspase 3, cleaved-caspase 3, Bax, and Bcl-2 was examined by western blotting. (d) Pro-caspase 3, cleaved-caspase 3, Bax, and Bcl-2 protein development gray value analysis histogram. Data indicate the mean ± SD (*n* = 5, *n*: number of the sample). Significant differences in different groups compared with the control group (without treatment with Ang II or *β*-sitosterol) are expressed as  ^*∗*^*P* < 0.05,  ^*∗*^ ^*∗*^*P* < 0.01, and  ^*∗*^ ^*∗*^ ^*∗*^*P* < 0.001. Significant differences in different groups compared with the Ang II-alone treatment group are expressed as ^#^*P* < 0.05, ^###^*P* < 0.01, and ^###^*P* < 0.001.

Flow cytometry was performed after annexin V-PITC/PI double staining to investigate whether *β*-sitosterol is involved in the apoptosis of A7r5 cells. As shown in Figures 3(a) and 3(b), treatment of A7r5 cells with Ang II decreased the proportion of apoptotic cells to 2.01% ± 0.34% (*P* < 0.001) compared with the control group. Meanwhile, the Ang II-alone treatment group showed significantly downregulated expression of pro-caspase 3, cleaved-caspase 3, and Bax, and upregulated expression of Bcl-2 (Figures 3(c) and 3(d)). However, apoptosis of Ang II-induced A7r5 cells increased after *β*-sitosterol treatment (*P* < 0.05). This effect of *β*-sitosterol was also confirmed by the upregulated expression levels of pro-caspase 3, cleaved-caspase 3, and Bax, as well as the downregulated expression of Bcl-2. These results indicated that *β*-sitosterol could promote the apoptosis of Ang II-induced A7r5 cells.

### 3.6. Inhibitory Effect of *β*-Sitosterol on the Autophagy of Ang II-Induced A7r5 Cells

**Figure 4:**
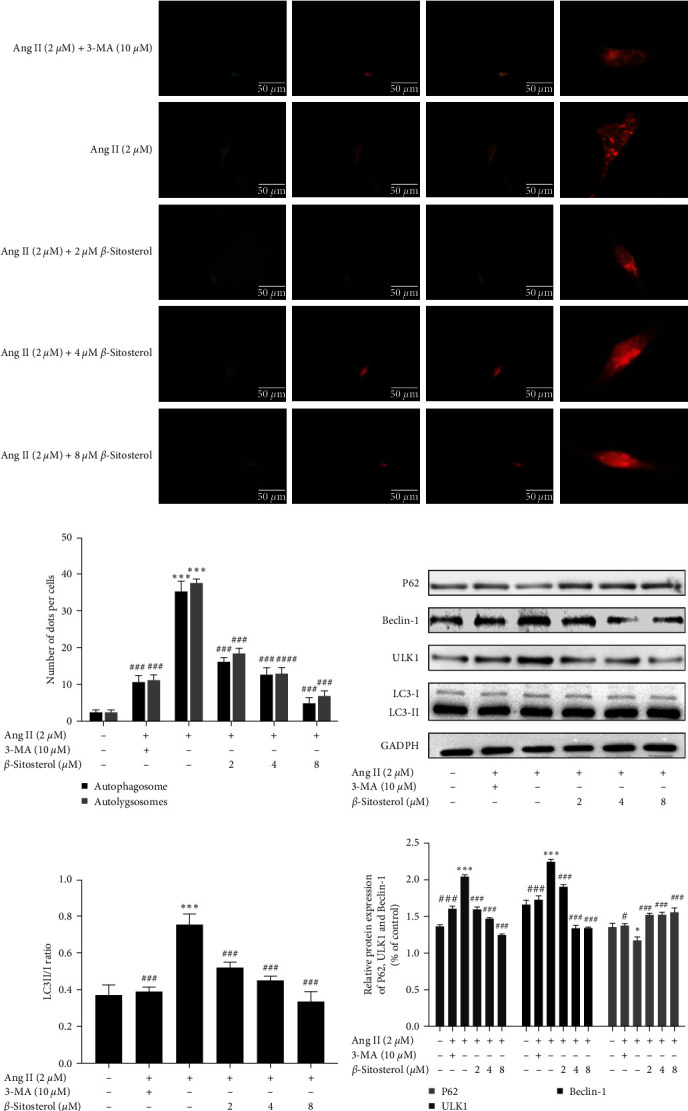
Effect of *β*-sitosterol on the autophagy of Ang II-induced A7r5 cells. (a) A7r5 cells were transfected with Ad-mCherry-GFP-LC3 and observed under a fluorescence microscope (400x), scale bar = 50 *μ*m. In the Ad-mCherry-GFP-LC3B assay, GFP only examines autophagosomes and mCherry examines autophagosomes and autolysosomes. The yellow dots (the combined effect of mCherry and GFP) indicate autophagosomes and the red dots indicate autolysosomes. (b) Number of dots per cell was analyzed. (c) Protein expression of P62, LC3, Beclin-1, and ULK1 was examined by western blotting. (d) The ratio of LC3-II/I of A7r5 cells were analyzed. (e) P62, Beclin-1, and ULK1 protein development gray value analysis histogram. Data indicate the mean ± SD (*n* = 5, *n*: number of the sample). Significant differences in different groups compared with the control group (without treatment with Ang II or *β*-sitosterol) are expressed as  ^*∗*^*P* < 0.05,  ^*∗*^ ^*∗*^*P* < 0.01, and  ^*∗*^ ^*∗*^ ^*∗*^*P* < 0.001. Significant differences in different groups compared with the Ang II-alone treatment group are expressed as ^#^*P* < 0.05, ^###^*P* < 0.01, and ^###^*P* < 0.001.

Abnormal autophagy in VSMCs is an important pathogenic process in the cardiovascular diseases. We determined whether *β*-sitosterol protects Ang II-induced A7r5 cells against excessive proliferation by anti-autophagy and examined changes in autophagic flux. The Ad-mCherry-GFP-LC3B assay of A7r5 cells showed that the number of yellow and red dots significantly increased after treatment with Ang II, and *β*-sitosterol reduced the number of yellow and red dots in Ang II-induced A7r5 cells in a concentration-dependent manner (Figures 4(a) and 4(b)). Meanwhile, the number of yellow and red dots also decreased in the 3-MA treatment group.

The expression of autophagy-related proteins (LC3, ULK1, P62, and Beclin-1) was analyzed using western blotting to confirm whether Ang II-induced autophagy at the cellular level was ameliorated by *β*-sitosterol. Western blot assays revealed increased LC3-II/I ratio, Beclin-1, and ULK1 protein expression levels, and decreased P62 expression levels in the Ang II-alone treatment group (Figures 4(c)–4(e)). However, this effect was reversed by treatment with *β*-sitosterol (2, 4, and 8 *µ*M) and 3-MA. The inhibitory effect of *β*-sitosterol was related to its concentration, and the most prominent inhibitory effect was observed at 8 *µ*M. The above results showed that *β*-sitosterol could inhibit autophagy in Ang II-induced A7r5 cells.

### 3.7. Reversal of *β*-Sitosterol Effects on Ang II-Induced A7r5 Cells Autophagy and Proliferation by Autophagy Activator Rapamycin

**Figure 5:**
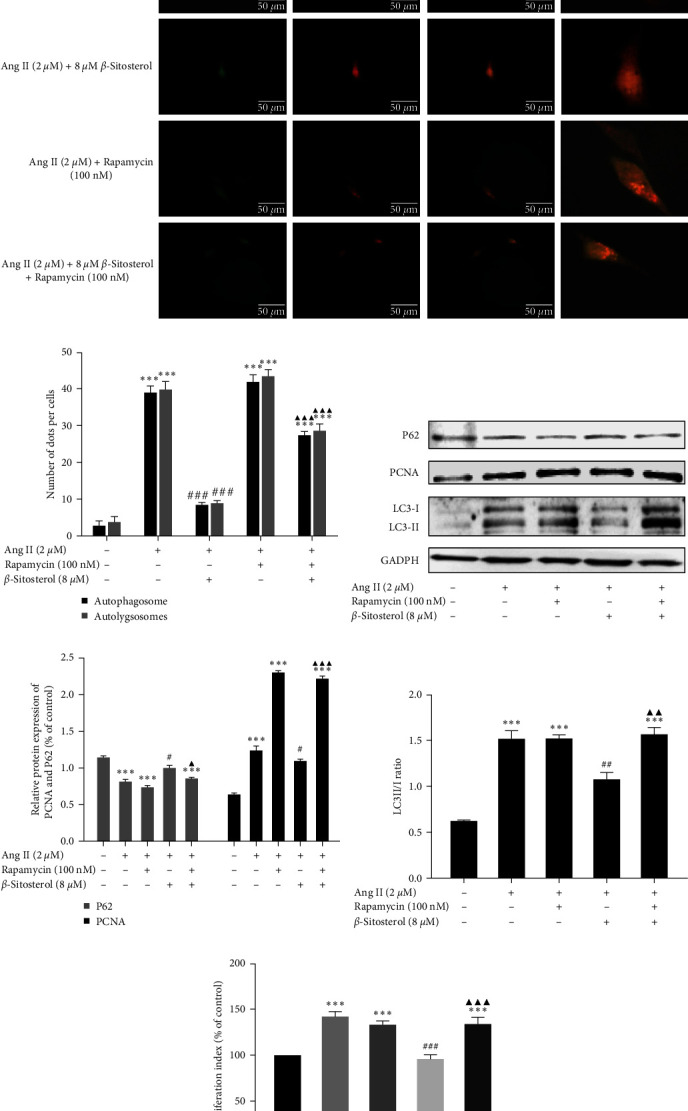
*β*-Sitosterol inhibited Ang II-induced A7r5 cell proliferation via downregulation of autophagy. (a) A7r5 cells were transfected with Ad-mCherry-GFP-LC3 and observed under a fluorescence microscope (400x), scale bar = 50 *μ*m. (b) The number of dots per cell was analyzed. (c) Protein expression of P62, LC3 and PCNA was determined using western blotting. (d) Gray value analysis histogram of PCNA and P62 protein development. (e) The LC3-II/I ratio in A7r5 cells was analyzed. (f) A7r5 cell proliferation was detected using CCK-8 assay. Data are presented as mean ± SD (*n* = 5, *n*: number of the sample). Significant differences in different groups compared with the control group (without treatment with Ang II or *β*-sitosterol) are expressed as  ^*∗*^*P* < 0.05,  ^*∗*^ ^*∗*^*P* < 0.01, and  ^*∗*^ ^*∗*^ ^*∗*^*P* < 0.001. Significant differences in different groups compared with the Ang II-alone treatment group are expressed as ^#^*P* < 0.05, ^###^*P* < 0.01, and ^###^*P* < 0.001. Significant differences in the rapamycin + Ang II + *β*-sitosterol combined treated group compared with the Ang II + *β*-sitosterol treatment group are expressed as ^▲^*P* < 0.05, ^▲▲▲^*P* < 0.01, and ^▲▲▲^*P* < 0.001.

Rapamycin is used to investigate the role of autophagy in the inhibitory effect of *β*-sitosterol on Ang II-induced proliferation of A7r5 cells. The *β*-sitosterol-induced reduction in Ang II-induced proliferation at 8 *µ*M was restored by treatment with 100 nM rapamycin (Figure 5(f)). Next, we wanted to confirm whether protein expression in Ang II-induced A7r5 cells could be influenced by rapamycin. Western blotting results showed that the decreased protein expression of PCNA in Ang II-induced A7r5 cells by 8 *µ*M *β*-sitosterol was increased by rapamycin administration (Figures 5(c) and 5(d)).

We also observed alterations in autophagic flux and changes in the protein expression of LC3 and P62. Treatment of Ang II-induced A7r5 cells with 8 *µ*M *β*-sitosterol resulted in a decrease in the LC3-II/I ratio, a reduction in the number of yellow and red dots, and an increase in P62 expression. Meanwhile, the LC3-II/I ratio increased and the expression of P62 decreased, while the number of yellow and red dots increased after cotreatment with *β*-sitosterol and rapamycin (Figures 5(a)–5(e)). These results showed that *β*-sitosterol inhibited the proliferation of Ang II-induced A7r5 cells by the downregulation autophagy.

## 4. Discussion

Clonal A7r5 embryonic rat thoracic aortic smooth muscle cells retain many characteristics of VSMCs and are thus considered a suitable model of VSMCs [22]. In this study, we have experimentally demonstrated that pretreatment with *β*-sitosterol exerts a remarkable protective effect on A7r5 cells by inhibiting Ang II-induced cell proliferation. Furthermore, for the first time, we identified that *β*-sitosterol regulates Ang II-mediated apoptosis, cell cycle, autophagy, and the contractile–synthetic phenotypic switch, which may participate in the antiproliferation process of *β*-sitosterol against Ang II in A7r5 cells.

Numerous studies have clearly demonstrated that Ang II, an important regulator of vascular remodeling, plays a crucial role in the occurrence and development of the cardiovascular diseases [23]. In this study, we found that Ang II induced the contractile–synthetic phenotypic switch and proliferation of VSMCs *in vitro*. Contractile and synthetic are two phenotypes of VSMCs. The transition from the contractile to synthetic form results in the proliferation of VSMCs in hypertension [24]. Uncontrolled proliferation, migration, and matrix synthesis of VSMCs are critical cytopathological links in the process of vascular remodeling in hypertension and atherosclerosis [25, 26]. In hypertension, vascular remodeling involves dynamic structural and morphological changes that narrow the blood vessels, thicken their walls, increase vascular resistance and pressure, and worsen hypertension and its complications [27]. Vascular remodeling in atherosclerosis exacerbates the vulnerability of atherosclerotic plaques, thereby increasing the risk of cardiovascular events [28]. In this study, we also found that different concentrations of *β*-sitosterol alleviated Ang II-induced A7r5 cell proliferation and inhibited the switch from a contractile to synthetic phenotype in A7r5 cells induced by Ang II. These findings suggest that *β*-sitosterol holds promise as a therapeutic option for vascular disorders, including hypertension and atherosclerosis.

The eukaryotic cell cycle consists of four phases: G0/G1, S, G2, and M. VSMCs generally exist in a nonproliferative state (G0/G1) under normal conditions. However, in case of blood vessel injuries, Ang II, PDGF-bb, and other stimulatory cytokines and growth factors can induce VSMCs to transform from the nonproliferative state (G0/G1) to the proliferative state (S phase) [29]. The present study found a similar cell cycle progression in Ang II-induced A7r5 cells. After treatment of A7r5 cells with Ang II, the proportions of cells in the G0/G1 phase decreased, and the proportions of cells in the S phase increased. The previous research has demonstrated that *β*-sitosterol possesses the ability to impede the proliferation of human leukemia cells by inducing cell cycle arrest at G2/M phases [30]. Our study found that *β*-sitosterol significantly decreased the proportion of A7r5 cells in the S phase and increased the proportion of A7r5 cells in the G0/G1 phase. The cell cycle is a complex process that involves multiple regulatory proteins [31]. Cyclin D1, an important cyclin involved in the cell cycle regulation, plays a key role in transforming the G1 phase into the S phase [32]. The upregulation of Cyclin D1 protein expression can significantly accelerate the transition from the G1 phase to the S phase, causing excessive cell proliferation and tumor formation [32–34]. PCNA is a nuclear protein involved in the S phase and is an accessory protein that mediates DNA polymerase replication of the DNA of cells [35, 36]. Blockade of the protein expression of Cyclin D1 and PCNA could significantly reduce the proliferation of aberrant VSMCs in certain pathological events [37]. In the present study, Ang II upregulated the protein expression of Cyclin D1 and PCNA in A7r5 cells, which aligns with the previous findings in VSMCs [20]. However, treatment with *β*-sitosterol reduced the protein expression of Cyclin D1 and PCNA in Ang II-induced A7r5 cells. These results suggest that *β*-sitosterol inhibits Ang II-induced A7r5 cell proliferation by blocking cell cycle progression.

Notably, there is strong evidence that Ang II can inhibit the apoptosis of mouse aortic smooth muscle cells and lead to vascular remodeling [38–40]. In the present study, A7r5 cells treated with Ang II exhibited a significantly lower proportion of apoptotic cells compared to the control group. The previous studies have shown that *β*-sitosterol induces apoptosis and inhibits the proliferation of ovarian cancer cells. Similarly, our study also showed that *β*-sitosterol can induce apoptosis in A7r5 cells stimulated by Ang II. Apoptosis is a form of programed cell death that effectively removes unwanted host cells to maintain homeostasis. Bcl-2 and Bax genes are important regulators of apoptosis in the apoptosis signal transduction pathway [41]. These genes have opposite roles in apoptosis regulation. Caspase 3, the most critical factor in apoptosis downstream of the caspase cascade, catalyzes the cleavage of specific proteins in cells [42]. Bcl-2 and Bax can activate caspase 3 to initiate cell apoptosis by releasing cytochrome c and other factors through the mitochondrial pathway. Cleaved-caspase 3 is an activated form of caspase 3 [43]. In the current study, 2 *µ*M Ang II inhibited the apoptosis of A7r5 cells primarily by downregulating the protein expression of pro-caspase 3, cleaved-caspase 3, and Bax, and upregulating the protein expression of Bcl-2. Similar results were reported in the other studies [44]. *β*-Sitosterol has been reported to promote apoptosis in human gastric cancer SGC 7901 cells and human leukemia U937 cells by upregulating the expression of cleaved-caspase 3 and Bax proteins, while downregulating the expression of Bcl-2 protein [12]. In the present study, treatment with *β*-sitosterol upregulated pro-caspase 3, cleaved-caspase 3, and Bax protein expression while downregulating Bcl-2 protein expression in Ang II-induced A7r5 cells. These results indicate that *β*-sitosterol partially alleviates Ang II-induced A7r5 cell proliferation by promoting apoptosis.

Autophagy has gained increasing attention as an evolutionarily conserved mechanism that is associated with a variety of cellular signaling pathways and plays a crucial role in the survival and function of VSMCs [45, 46]. It has been reported that autophagy contributes to Ang II-induced VSMC hypertrophy through an AT1R/RhoA/Rho kinase-dependent mechanism [7]. In this study, we found that Ang II induces autophagy in A7r5 cells. Additionally, *β*-sitosterol has the potential to enhance nonsmall cell lung cancer treatment by inhibiting autophagy [47]. Our study showed that *β*-sitosterol inhibits Ang II-induced autophagy in A7r5 cells. ULK1 is a gathering point for multiple signaling pathways that regulate autophagy initiation [48]. A previous study has reported that ULK kinase promotes the recruitment of Beclin-1 to the endoplasmic reticulum, thereby driving the formation of autophagosomes [49]. Beclin-1, a class III phosphatidylinositol 3-kinase component, plays a significant role in transport processes mediated by the inner membrane, including autophagosome transport [50]. Additionally, overexpression of Beclin-1 increased the levels of ATG4, ATG5, and LC3-II proteins in tongue squamous cell carcinoma cell lines [51]. LC3-II, the lipidated form of LC3, is an autophagosomal marker in mammals and is often used to study autophagy in the cardiovascular diseases. P62 acts as a bridge between LC3 and ubiquitinated proteins during autophagosome formation. It gets selectively encapsulated into autophagosomes and subsequently degraded by proteases in autolysosomes, leading to an inverse correlation between P62 expression and autophagic activity [52]. In our study, Ang II induced autophagy in A7r5 cells primarily by increasing the ratio of LC3II/I and by enhancing the protein expression of Beclin-1 and ULK1, while downregulating the expression of P62. Similar results have been reported in the previous studies on cardiomyocytes [53]. *β*-Sitosterol supplementation significantly inhibited autophagy in Ang II-induced A7r5 cells, with a concomitant decrease in LC3, Beclin-1, and ULK1 expression, and a concurrent upregulation of P62 expression in A7r5 cells. We found, for the first time, that *β*-sitosterol could reverse Ang II-induced autophagy in A7r5 cells.

Several studies have demonstrated that inhibition of autophagy contributes to a reversible reduction in cell proliferation in various cell lines, including VSMCs and macrophages [54]. Cortistatin protects VSMCs from Ang II-induced proliferation by inhibiting autophagy by regulating SSTR3 and SSTR5 receptors [10]. Overexpression of miR-145 ameliorates the proliferation and migration of TGF *β*1-induced VSMCs by inhibiting autophagy activation [55]. In the present study, *β*-sitosterol significantly inhibited Ang II-induced autophagy and proliferation of A7r5 cells, which could be reversed by the autophagy activator rapamycin. Thus, we speculate that the inhibitory effect of *β*-sitosterol on the excessive proliferation of Ang II-induced A7r5 cells is also related to the regulation of autophagy. However, considering gene expression profiles from Ang II-treated A7r5 cells which are used in the different laboratories, the variations derived from the different cell types should be of concerned. There are also differences between different vascular smooth muscle cell lines and experimental conditions. Therefore, more vascular smooth muscle cell lines should be employed for the mechanistic investigation. The siRNA, protein inhibition, and in vivo experiments are still needed to clarify the inhibitory effect of *β*-sitosterol on the excessive proliferation of Ang II-induced A7r5 cells.

## 5. Conclusion

This study found for the first time that *β*-sitosterol inhibited Ang II-induced proliferation of A7r5 cells by inducing cell cycle arrest, promoting apoptosis, inhibiting autophagy, and suppressing the contractile-synthetic phenotypic switch. These findings indicate that the administration of *β*-sitosterol has broad developmental potential as a therapeutic strategy to prevent cardiovascular diseases.

## Data Availability

The data that support the findings of this study are available from the corresponding author, Lu Q, upon reasonable request.
